# Development of a professional advancement model for perfusionists

**DOI:** 10.1051/ject/2024025

**Published:** 2024-12-20

**Authors:** Adam K. Blakey

**Affiliations:** 1 Virginia Commonwealth University Health System 1250 East Marshall Street Richmond VA 23298 USA

**Keywords:** Professional Advancement Model, Perfusionists, Turnover, Retention

## Abstract

*Background*: Improvement in professional advancement opportunities may reduce turnover and improve retention for perfusionists. However, the current literature lacks examples of professional advancement models (PAMs) for perfusionists. *Methods*: This review looks at examples from other healthcare fields to provide the rationale and develop a framework for such a model. *Results*: The review results led to the development of a point-based PAM that included four levels: perfusionist I, II, III, and IV. Each level is associated with its own point requirement, experience level, and salary increase. Points can be acquired through four defined categories. *Conclusion*: Perfusion programs needing professional advancement can use these results as a foundation for implementing a PAM for perfusionists.

As of December 31, 2023, over 4,800 perfusionists were employed in the United States [1]. The United States Bureau of Labor Statistics does not measure perfusionist data [2]. Given the lack of government data sets to assess perfusionists, a recent survey asked perfusionists questions about perceptions and the variables related to vacancy and turnover rates, as well as strategies that could be implemented to improve them [3]. The survey found that the vacancy and turnover rates were 12.3% and 14.7%, respectively, and “career advancement opportunities” were identified as a prominent strategy for decreasing vacancy and turnover [3].

To mitigate the high turnover and retention rates for perfusionists, leaders must find ways to meet the needs of their staff perfusionists. In other healthcare fields, such as nursing and those belonging to the group of healthcare professionals known as advanced practice providers (APPs), professional advancement models (PAMs) implemented by health systems have had a positive impact on retention rates [4]. APP is a blanket term applied to the following professions in the United States: Nurse practitioners (NPs), Clinical Nurse Specialists (CNSs), certified registered nurse anesthetists (CRNAs), physician assistants (PAs), and certified nurse midwives (CNMs). Other healthcare roles demonstrating PAM development include paramedics, sonographers, pharmacists, and respiratory therapists [5–8]. Additionally, while not a professional advancement model per se, physicians employed by academic medical centers are promoted through the ranks from assistant professor to full professor [9].

There is no published literature on developing a PAM for perfusionists. This literature review will first define what a PAM is for healthcare employees. Next, it will examine the rationale for creating a PAM using evidence from other healthcare fields regarding outcomes and implementation success. Then, it will explore the specific needs and framework associated with developing a PAM using evidence from similar healthcare fields. Lastly, the discussion will review the strengths and weaknesses of the body of literature and apply the framework findings to fit the requirements of a perfusionist PAM.

## Literature review

### Defining a professional advancement model

A professional advancement model (PAM), also known as a clinical ladder or clinical advancement program, is a health system program that serves as a career advancement framework for healthcare professionals by offering incentives and promotions for performance above the job’s basic requirements [10]. Benner originally described a nursing PAM commonly used as the basis of framework developments today, which applied five proficiency levels to nursing based on skill and knowledge acquirement: novice, advanced beginner, competent, proficient, and expert [11]. Promotion to each level is obtained sequentially by meeting increasingly difficult metrics defined by each PAM and is typically accompanied by a salary increase or other benefits [4, 10, 12].

### Rationale for creating a professional advancement model

PAMs effectively reduce yearly turnover among APPs, reducing 13.8% to 5% over four years after implementation [4]. In the same study, participants in the PAM program were found to have broad support for the process. Similarly, another study showed a decrease in organizational APP turnover rate from 22% before PAM implementation to 5% after [13].

A survey of 162 nurses who were part of a clinical advancement program for an extensive academic health system found overwhelming positivity about the impact on a nurse’s career [10]. Most respondents agreed that the advancement program helped them grow professionally and positively impacted employee satisfaction and retention of nurses. Another survey of 157 APPs found that most respondents either agreed or strongly agreed that an organizational PAM made them more interested in seeking professional opportunities and strengthened their clinical practice [14].

Implementing a PAM for APPs helps increase APP visibility at the organizations where they work and publicly through increased journal publications and conference presentations [13]. In the same study, after implementing a PAM for APPs, the authors found that abstract submission to a regional conference more than doubled from 20 in year one to 45 in year three [13].

### Framework for developing a professional advancement model

McComiskey et al. presented the APP PAM implementation in their organization [4]. The authors first surveyed APPs in the organization’s desire to seek professional advancement opportunities. The PAM consisted of a four-tier model: Level I, Level II, Senior I, and Senior II. Requirements for Level I included entry-level skills and less than one year of experience. Level II can be obtained by having more than one year of experience and obtaining specialty certifications. Senior I must have an excellent annual performance review before applying. The Senior I applicant must meet well-defined clinical and leadership metrics, such as leading a quality improvement initiative or publishing an article. In addition to the requirements of the lower levels, Senior II must also demonstrate more advanced leadership and clinical metrics, such as holding a leadership position in a professional organization and grant proposal submission. Implementation of the PAM consisted of Levels I and II automatically being given to APPs who met the criteria. For Senior I and II, participants must submit applications to a PAM committee consisting of APPs who evaluate each applicant and either approve or deny the promotion. Senior I and II both experienced a 5% salary increase.

Warman et al. describe redesigning a clinical advancement program for nurses created eight years prior [10]. The redesign included a two-tier model: Levels II and III. The framework consisted of the following categories in which the nurses must meet specific metrics: Exemplary professional practice, transformational leadership, structural empowerment, and new knowledge, innovation, and improvements. A point system was created where points were assigned to specific tasks within each category. For example, enrollment in a master’s program, becoming a preceptor, giving a poster presentation, and journal club participation are examples of tasks within each category. Level III had a higher point requirement than level II, and minimum point requirements within each category were required. To be eligible for Level II, nurses must have been employed for at least one year and exceeded performance evaluation standards. Advancing to Level III requires employment in their clinical area for at least five years and must reach Level II before applying for Level III. Participants must submit their portfolios to a committee consisting of nursing leadership, where they either approve or deny the promotion. An appeals process is also in place for those who feel they were wrongly denied. Level II experienced a 5% salary increase, and Level III experienced a 10% increase.

Ko and Yu describe a nursing clinical ladder implementation that stresses the importance of presenting leadership and administration with estimated costs and getting approval before beginning the work [12]. After approval, the article describes the implementation of a four-level PAM. A minimum of one, three, five, and seven years of experience were required for promotion to levels one, two, three, and four, respectively. Level one did not have any additional requirements. Level two must have evaluated well, participated in nursing education programs and committee activities, and taken various learning courses. In addition to the requirements of Level two, Level three required a bachelor’s degree, excellent performance evaluations, and obtained certifications related to nursing. Level four required all of the accomplishments of lower levels, a master’s degree, and a research publication. Rewards for each level were tuition reimbursement, an additional two days of vacation time, and promotion to unit manager for levels two, three, and four, respectively.

A five-level conceptual clinical ladder model for PAs was developed by Boyd et al. [15]. In this conceptual model, an applicant could obtain points across the four categories: clinical practice development, clinical leadership, clinical scholarships, and clinical service. Specific activities include enrollment in a specialty certificate program, membership in a professional PA organization, publication in a scholarly journal, and participation in a non-clinical community-based volunteer project. The proposed requirements for each level were none for PA I, three years of experience plus ten criteria points for PA II, five years of experience plus 12 criteria points for Senior PA, seven years of experience plus 15 criteria points for Chief PA, and eight years of experience plus 15 criteria points for executive PA.

The research by Burket et al. describes the application and renewal process for a clinical ladder for nurses [16]. Applicants must first fill out an application that includes, among other things, a self-evaluation and a demographic profile. Applicants must then select a coach and submit their application along with a curriculum vitae (CV), three letters of recommendation, and a letter of intent for which level is being applied. Documentation supporting the accomplishments that qualify the applicant for the level to which they are applying is also required. A committee reviews the material and then formally interviews the applicant, resulting in either a promotion or denial. To maintain the current level on the ladder, nurses must renew their level by maintaining at least 90% of the criteria required for promotion to that level [16].

## Discussion

### Application of findings to create a perfusionist PAM

Using the findings from the literature review, this discussion will be separated into the following categories: Institutional support, gauging perfusionist interest, PAM committee and application process, level and promotion criteria, and follow-up metrics.

#### Institutional support

Identifying the stakeholders affected by a PAM is an essential first step [15]. Implementing a PAM will be costly, so administrative and clinical leaders must agree it is a good investment [12, 13]. For example, a PAM is typically signified by a salary increase when promoted from one level to another, ranging from 5% to 30%, depending on the level acquired [4, 13, 15]. Leaders should be able to estimate the additional cost of PAM implementation based on their employees’ salaries. There are costs associated with high turnover and the hiring process, including indirect costs that may not be obvious [17]. PAM implementation in other healthcare fields has been shown to reduce staff turnover, so a connection can be made that a PAM may be in the best financial interest of the healthcare organization [4, 13]. Looking past the financial considerations, hospital leaders may find value in increased academic standing from more publications due to PAM implementation [13].

#### Gauging perfusionist interest

McComiskey et al. surveyed APPs during the PAM planning phase to get their opinions on a PAM for their institution [4]. A similar survey can be conducted among perfusionist staff. Ideally, the survey will assess job satisfaction and whether perfusionists desire a PAM to promote career advancement. The feasibility of such a survey should be reasonable, considering the small size of most perfusion departments. According to a survey, the size of a perfusionist team can range from 1 to 37, but most respondents came from a team with only three perfusionists [3].

#### PAM review committee and application process

Establishing a committee with decision-making authority concerning the approval or denial of the promotion is recommended. For example, McComiskey et al. describe the committee for an APP PAM as having members of the CNS, NP, PA, and CRNA teams [4]. Additionally, supervisors and peers of the applying team members were not allowed to participate. While not mentioning restrictions on who can review an application, Warman et al. describe a committee for a nursing clinical ladder, which comprises nurses who have advanced to either level II or level III of their ladder [10].

It is important to note that APP and nursing programs at institutions are much larger than most perfusion departments. For example, the study by Warman et al. describes a health system where 3,000 nurses are employed [10]. Similarly, McComiskey et al. state that their health system employs 300 APPs [4]. With the small number of perfusionists employed in a health system, it is not feasible for a perfusionist PAM committee to not allow peers or supervisors to review a PAM application without excluding perfusionists entirely from the process. Depending on the scope and reporting structure of perfusion departments within a health system, including a perfusionist leader from both adult and pediatrics, a cardiac surgeon, a cardiac anesthesiologist, and an administrator on the PAM committee may be reasonable.

Applications for advancement should be standardized. The applicant should submit to the committee a CV, letters of recommendation, the level at which they intend to apply, and supporting documents [16]. Additionally, application windows are a reasonable addition to the process. For example, McComsikey et al. describe their application process as opening every six months for a two-week window [4]. Following this two-week window, a two-week review process is conducted, during which the applicants are either approved or denied promotion.

#### Level and promotion criteria

***Level definition, point system, and experience requirement***. Evidence supports the use of between three and five levels where clinicians can be promoted, each supporting the first level as an entry-level position [4, 10, 12, 15]. Similarly, the literature review supports placing experience requirements on each level [4, 10, 12, 15]. Additionally, a point system where applicants obtain points toward promotion by completing activities within specific categories is frequently described in the literature [10, 15]. Table 1 displays the experience and points required to progress through the levels. The proposed perfusionist PAM will consist of four levels: Perfusionist I, II, III, and IV. The required experience level will be three, five, and seven years of experience for levels II, III, and IV, respectively. Perfusionist I requires no experience as it is an entry-level position.

**Table 1:** Levels, points, and experience required.

Perfusionist I	Perfusionist II	Perfusionist III	Perfusionist IV
0+ Years	3+ years	5+ years	7+ years
	10 points	12 points	15 points

***Point categories, activities, and quantities***. When referring to points accumulated as part of the promotion process, the accrual should be limited to points acquired within the past 24 months [15]. The perfusionist PAM categories and associated activities and points are listed in Table 2. The following categories were created: Leadership, Clinical Excellence, Education, and Service. The number of points awarded was determined by the work by Boyd et al. as well as the opinion of this author [15].

**Table 2:** Professional advancement model categories, activities, and points.

Leadership	Points
Member of a professional organization	1
Institutional committee/task force member	2
Involvement in department initiatives	1
Leadership position in a professional organization	2
Leadership development course	1
Other activity with approval	1
Clinical Excellence	
Skills workshop participation	1
Assist with quality improvement initiative	2
Conference attendance	1
Journal club participation	1
Article publication	2
Book chapter publication	2
Research study participation	2
Other activity with approval	1
Education	
Degree advancement – Masters	2
Degree advancement – Doctorate/PhD	2
Provide interdisciplinary lecture	1
Provide department lecture	1
Poster presentation	1
Podium presentation	2
Other activity with approval	1
Service	
Assists with new staff orientation	1
Holds clinical or adjunct faculty appointment	1
Community-based volunteer project	2
Clinical Instructor	1
Student clinical coordinator	1
Simulation facilitator	1
Manuscript peer-reviewer	2
Other activity with approval	1

*Leadership.* Six activities are included within the leadership category. The literature review supports being a member of a professional organization, a member of an institutional committee/task force, and having a leadership role in a professional organization as categorical points [4, 10, 15]. Warman et al. included attending a leadership development course, while Boyd et al. and McComiskey et al. also included being an active member in department initiatives and other activities with prior approval [4, 10, 15].

*Clinical Excellence.* Seven activities are included within the clinical excellence category. Boyd et al. support the addition of skills workshop participation, while Warman et al. and McComiskey et al. list journal club participation and assisting quality improvement initiatives as activities [4, 10, 15]. Scholarly works such as book chapter publications and article publications are included in other PAMs [4, 15]. Conference attendance and research study participation are found in other PAMs and included in the clinical excellence category [4, 10, 15]. Lastly, other activities with approval are also included.

*Education.* Six activities are included within the education category. Boyd et al. and Warman et al. include interdisciplinary and department-specific lectures as educational activities worth points [10, 15]. Poster and podium presentations at conferences are also supported by other PAMs [4, 10]. In addition to including a Doctorate/PhD degree advancement activity, the perfusionist PAM includes a degree advancement to a master’s degree [4]. The importance of including a Master’s degree advancement option is not to exclude a large portion of perfusionists who would seek to advance their degree. For example, a recent survey of female perfusionists found that most females have either a bachelor’s or certificate degree [18]. Lastly, additional activities are also included with the proper approval.

*Service.* Seven activities are included within the service category. The work by McComiskey et al. supports assisting new staff with orientation and holding a clinical or adjunct faculty appointment [4]. Boyd et al. and Warman et al. support including being a clinical instructor and providing community volunteer work [10, 15]. Being a simulation facilitator and a manuscript peer reviewer were also included [4, 10]. Being a student clinical coordinator for perfusionist students is also included, as well as other activities that may be outside the list.

#### Follow-up metrics

Promotion ideally should be challenging but ultimately still attainable. For example, research by McComiskey et al. showed that over ten years, a PAM for APPs had a promotion rate of 75% [19]. Upon surveying the clinicians who had applied to the program, 52% agreed that the promotion was difficult to attain, while 62% agreed that the content needed to be promoted was fair [19]. Likewise, Arthur et al. demonstrated an overall program completion rate for all levels of 47%, 55%, and 51% for years one, two, and three, respectively [13].

Salary increases should accompany the promotion, ranging from 5% to 30% for other healthcare fields, depending on the promotion level [4, 13, 15]. Based on the difficulty of obtaining each promotion level, it is reasonable to have 5%, 10%, and 15% for Perfusionist II, III, and IV, respectively.

## Limitations

A notable limitation of the literature review is that there is no evidence of a PAM specifically for perfusionists. While this limitation concerns generalizability, it also presents an opportunity to learn from other healthcare fields that have implemented PAMs in their workplaces. The type of institution that implements a PAM may also be a limitation. For example, much of the literature focuses on academic medical centers in the United States [4, 6, 10, 13, 14]. The results of this review may not be generalizable to perfusionists employed by such entities as private institutions or physician groups or to perfusionists residing in countries other than the United States.

The point system criteria and salary increases are based on published literature findings but are unlikely to be universal among all perfusionist programs looking to implement a PAM. Each health system is unique, and the financials of different programs and the emphasis on different categories of points will also likely differ as assigning point values is difficult. Warman et al. wrote, “The actual task of assigning points in the categories was more challenging than originally thought” [10]. Furthermore, Slagle et al. performed a literature review looking at nursing clinical ladders and emphasized the heterogeneity in the literature regarding the framework development of such programs [20]. Lastly, another limitation is that this perfusionist PAM was a conceptual development and stopped short of the implementation process.

## Future considerations

Other healthcare professional organizations champion using PAMs by disseminating examples and information that members can use [21, 22]. Professional perfusionist organizations, such as the American Society of Extracorporeal Technology (AmSECT), could be pivotal in advancing PAM development for perfusionists. For example, a PAM committee within a professional organization could be established to create and disseminate policy documents that guide perfusion programs aiming to implement PAMs across the profession. By fostering and setting industry standards, professional organizations could help to ensure the adoption of PAMs, which could benefit perfusionists and the entire profession.

## Conclusion

Improving career advancement opportunities for perfusionists is a possible strategy to reduce vacancy and turnover [3]. A professional advancement model (PAM) for other healthcare fields, such as advanced practice providers (APPs) and nursing, has been shown to reduce turnover, improve job satisfaction, and improve clinician visibility through publications [4, 10, 13, 14]. Literature detailing a development process for a perfusionist professional advancement model (PAM) is nonexistent.

Using literature from other healthcare fields, a perfusionist PAM was created. The first steps are to understand the costs associated with a PAM and garner institutional support, followed by gauging staff perfusionist interest in such a program. A PAM committee in charge of approving or denying promotions and the application process must also be established. The proposed perfusionist PAM consisted of four levels: Perfusionist I, II, III, and IV, with perfusionist I being an entry-level position. A point system was developed as metrics to meet for promotion, applied across the following four categories: Leadership, clinical excellence, education, and service. A combination of points and experience was used when determining eligibility for promotion.

Perfusion programs needing a tool to increase retention and decrease turnover should consider developing a PAM. The results of this literature review led to the development of PAM for perfusionists that can serve as a framework for programs looking to develop their model.

## Data Availability

All available data is incorporated into the article.
